# The Use of Sodium Bicarbonate in the Treatment of Acidosis in Sepsis: A Literature Update on a Long Term Debate

**DOI:** 10.1155/2015/605830

**Published:** 2015-07-30

**Authors:** Dimitrios Velissaris, Vasilios Karamouzos, Nikolaos Ktenopoulos, Charalampos Pierrakos, Menelaos Karanikolas

**Affiliations:** ^1^Internal Medicine Department, University Hospital of Patras, 26500 Rion, Greece; ^2^University of Patras School of Medicine, 26500 Rion, Greece; ^3^Intensive Care Department, Brugmann University Hospital, 1030 Brussels, Belgium; ^4^Department of Anesthesiology, Washington University School of Medicine, St. Louis, MO 63110, USA

## Abstract

*Introduction*. Sepsis and its consequences such as metabolic acidosis are resulting in increased mortality. Although correction of metabolic acidosis with sodium bicarbonate seems a reasonable approach, there is ongoing debate regarding the role of bicarbonates as a therapeutic option. *Methods*. We conducted a PubMed literature search in order to identify published literature related to the effects of sodium bicarbonate treatment on metabolic acidosis due to sepsis. The search included all articles published in English in the last 35 years. *Results*. There is ongoing debate regarding the use of bicarbonates for the treatment of acidosis in sepsis, but there is a trend towards not using bicarbonate in sepsis patients with arterial blood gas pH > 7.15. *Conclusions*. Routine use of bicarbonate for treatment of severe acidemia and lactic acidosis due to sepsis is subject of controversy, and current opinion does not favor routine use of bicarbonates. However, available evidence is inconclusive, and more studies are required to determine the potential benefit, if any, of bicarbonate therapy in the sepsis patient with acidosis.

## 1. Introduction

Sepsis and septic shock constitute an important cause of morbidity and mortality in the critically ill patient. Metabolic acidosis, including lactic acidosis, is part of the underlying pathophysiology in sepsis and is related to poor prognosis.

The treatment of metabolic acidosis is based on control of the underlying pathophysiologic process and reversal of organ dysfunction. Etiologic treatment is essential in metabolic acidosis, but optimization of oxygen delivery to tissues and reduction of tissue oxygen demand through sedation and mechanical ventilation are parts of the therapeutic strategy. Severe acidemia in sepsis contributes to hemodynamic instability, which is the result of reduced myocardial contractility, arterial vasodilation, and impaired responsiveness to catecholamines. As the effect of alkaline therapy on vasopressor requirements and hemodynamic profile in severe acidosis (pH ≤ 7.15) is unknown, current Surviving Sepsis Campaign guidelines recommend against treatment with bicarbonates. Despite lack of data on the effect of bicarbonate therapy, many intensivists attempt to alkalinize blood with intravenous administration of sodium bicarbonate as part of the treatment of sepsis. However, the benefit of bicarbonate administration in metabolic acidosis in sepsis is controversial and remains a matter of debate in clinical practice.

The aim of this review is to evaluate and summarize the published literature regarding the indications and benefits of sodium bicarbonate administration in septic patients with metabolic acidosis.

## 2. Methods

We conducted a literature search in the MEDLINE database (January 1980 to March 2015), using the combination of the terms “sepsis,” “metabolic acidosis,” and “bicarbonates.” The literature search included all type of articles, and the bibliography from all relevant extracted manuscripts was reviewed for identification of additional related references.

All identified manuscripts, including reviews, case series, and case reports, were evaluated for relevance, and articles deemed pertinent, current, and representative were included in this review. Publications in languages other than English and without abstract in English were excluded. Two authors evaluated the abstracts of all identified articles (DV and MK) and the full text of letters and case reports. Differences of opinion were resolved by reaching consensus after discussion.

## 3. Results

The PubMed literature search was conducted in March 2015 and yielded 51 publications.

A prospective, randomized, blinded, crossover study published in 1990 by Cooper et al. assessed bicarbonate administration on 14 intensive care unit (ICU) patients with metabolic acidosis and increased arterial lactate. In this study, each patient received sodium bicarbonate (2 mmol/kg infused over 15 minutes) and equimolar sodium chloride sequentially, in random order. After sodium bicarbonate administration, arterial pH, serum bicarbonate, and arterial blood partial CO_2_ pressure (PaCO_2_) increased, while plasma ionized calcium decreased. Both sodium bicarbonate and sodium chloride transiently increased pulmonary capillary wedge pressure and cardiac output, but mean arterial pressure did not change and hemodynamic responses to sodium bicarbonate and sodium chloride were the same. This study concluded that the hemodynamic and cardiovascular response to catecholamines did not improve with sodium bicarbonate administration [1].

Another prospective, randomized, blinded, crossover study published by Mathieu et al. in 1991 assessed bicarbonate administration (1 mmol/kg) for correction of acidemia in ten patients with lactic acidosis, by measuring hemodynamic variables and evaluating tissue oxygenation. Each patient received sequentially sodium bicarbonate and sodium chloride in random order. Sodium bicarbonate administration resulted in increased arterial and venous blood pH, but hemodynamic variables did not improve, and tissue oxygenation, as measured by oxygen delivery, oxygen consumption, oxygen extraction, and transcutaneous oxygen pressure, did not change. The study concluded that sodium bicarbonate did not significantly change hemodynamic profile or worsen tissue oxygenation [2].

Another prospective clinical trial published in 2008 by Fang et al. assessed the effects of resuscitation with 5 mL/kg of three different solutions (normal saline versus sodium chloride 3.5% versus sodium bicarbonate 5%) on cardiac function in 94 patients with severe sepsis and showed that although mean arterial pressure and cardiac output improved faster in the sodium bicarbonate group, there was no significant difference between the three groups with regard to pH, lactic acid, cardiac output, heart rate, or 28-day mortality [3].

A prospective, observational study by Noritomi et al. in 2009 in ICU patients showed that acidosis in survivors was corrected as a result of decreased lactate and strong ion gap levels, whereas metabolic acidosis did not improve in nonsurvivors [4]. A retrospective study published in 2010 by El-Solh et al. compared 36 patients with septic shock versus a group of 36 patients matched for age, site of infection, and predicted mortality who did not receive bicarbonate therapy and showed that duration of the need for mechanical ventilation and ICU length of stay were reduced in patients who received bicarbonates, but the median time until reversal of shock and 28-day mortality did not differ between groups [5].

Similarly, a prospective, multicenter observational study on 200 patients with severe acidemia (pH < 7.20) from 5 ICUs in France showed that bicarbonate use varied between 5% and 55% of patients between institutions, bicarbonate administration depended on the medical center, rather than the mechanism of acidemia, and there was no association between bicarbonate administration and outcome [6].

Another retrospective analysis by Kim et al. in 2013 analyzed 103 patients with lactic acidosis of multiple causes, including sepsis. Multivariate logistic regression showed that SOFA score and sodium bicarbonate administration were independent factors associated with higher mortality [7]. According to this study, lactic acid levels should be checked to evaluate the course of metabolic acidosis in sepsis patients, particularly those with high anion gap, as there is concern that bicarbonates could increase mortality.

A prospective randomized, double-blind, controlled clinical trial by Chen et al. in 2013 assessed the use of sodium bicarbonate in two stages for treatment of lactic acidemia due to hypoperfusion in 65 critically ill patients with septic shock and concluded that bicarbonate therapy significantly improved cardiac index, oxygen delivery, and mixed venous oxygen saturation and resulted in lower incidence of multiple organ dysfunction, shorter duration of mechanical ventilation, shorter ICU and hospital stay, and lower mortality [8]. Of note, the study by Chen et al. was published in Chinese but comes with a very detailed abstract in English, and we chose to include it in our review because its findings are markedly different compared to most other published clinical studies on the subject. Data from clinical studies evaluating the use of sodium bicarbonate in sepsis patients with acidosis are summarized in Table 1.

A survey published by Kraut and Kurtz in 2006 clearly shows the uncertainty among clinicians as to whether or not bicarbonates should be given to patients with metabolic acidosis. In this survey, 86% of nephrologists would give bicarbonate for treatment of lactic acidosis, compared to only 67% of intensivists. The authors concluded that the observed variability between physicians shows the need for further studies in order to generate data that could support evidence-based guidelines [9].

With regard to reviews, opinion articles, and guidelines, Levraut and Grimaud in 2003 reported a theoretical approach to metabolic acidosis, which should be differentiated to “mineral metabolic acidosis” or “organic metabolic acidosis.” The authors concluded that “mineral acidosis” is not related to failure of energy metabolic pathways and could be treated with bicarbonate administration to improve pH, whereas “organic acidosis” is evidence of severe underlying metabolic distress, and management is based on addressing the cause of acidosis, rather than correction of the acid-base imbalance [10]. Similarly, a 2004 publication by Cariou et al. summarized the recommendations presented in 2003 based on experts' opinion from 11 international organizations under the auspices of the Surviving Sepsis Campaign. These guidelines do not recommend bicarbonate administration as therapeutic option to improve hemodynamic status or reduce vasopressor requirements in sepsis patients with lactic acidosis and pH > 7.15 [11]. Similarly, a review article by Boyd and Walley in 2008 agreed with the Surviving Sepsis guidelines, recommended against bicarbonate use for pH > 7.15, and went even further, recommending a lower threshold for treatment at pH ≤ 7.0. These authors also suggested that bicarbonate-based replacement fluid is preferred to citrate infusion for continuous renal replacement therapy [12]. Last, the Surviving Sepsis Campaign guidelines in 2010 proposed a grade 2B recommendation for not using sodium bicarbonate therapy in sepsis patients for the purpose of improving hemodynamic status or reducing vasopressor requirements in hypoperfusion-induced lactic acidemia with pH ≥ 7.15 [13].

With regard to experimental data, dog experiments conducted in the 1970s suggest that plasma bicarbonate concentration influences the apparent volume of distribution of bicarbonate so that when bicarbonate is administered, the proportion of bicarbonate passing into the intracellular space increases with decreasing bicarbonate concentration [14]. A study by Valenza et al. in 2012 in normoxic, normotensive rats showed that pH decreased with infusion of lactic acid and rose with bicarbonate administration, but the effect of bicarbonate infusion on pH was different with persistent versus transient acid load [15]. An earlier study by Arieff et al. showed that bicarbonate administration did not improve hemodynamic parameters or reduce mortality in an animal model of lactic acidosis [16]. However, an experimental study by Benjamin et al. on dogs subjected to hemorrhagic shock showed that bicarbonate administration resulted in moderate increase of blood pressure, cardiac index, oxygen delivery index, and oxygen consumption index, but this benefit was not better compared to administration of hypertonic saline [17]. Other in vitro experiments performed by Goldsmith et al. in 1997 measured intracellular pH changes in leucocytes from healthy volunteers using a fluorescent intracellular dye and described deleterious effects after alkalinization of the extracellular fluid by giving sodium bicarbonate [18]. In addition, an in vitro study on human hepatocytes showed that bicarbonate administration can decrease intracellular pH, and the change in intracellular pH depends on pCO_2_ changes in the fluid surrounding the cells [19]. However, a newer study from Denmark evaluated intracellular pH of muscle tissue in healthy volunteers and showed that bicarbonate administration attenuated the reduction of intracellular pH induced by exercise [20]. Experimental data related to the use of bicarbonates in sepsis or acidosis are summarized in Table 2.

## 4. Discussion

Sepsis is a serious, potentially fatal disease commonly seen in intensive care units all over the world. Mortality rate is high (36.7%) in sepsis patients with hypotension only or with elevated lactate ≥ 4 mmol/L only (30%) but is even higher (46.1%) in patients with both hypotension and lactate ≥ 4 mmol/L. Metabolic acidosis is considered a sign of severity of the underlying disease process, and identification of the cause of acidosis is essential in order to initiate timely appropriate therapy. Causes of metabolic acidosis include sepsis, cardiogenic shock, severe hypoxemia, hepatic failure, and intoxication. Most of these conditions share similar pathogenic mechanisms, including reduced oxygen delivery to cells and impaired oxygen consumption in cell mitochondria, yet some conditions are due to more complex derangements. Lactic acidosis in sepsis is a complex process and the associated severe acidemia has important clinical consequences, including hemodynamic instability due to reduced left ventricular contractility, diastolic dysfunction and right ventricular failure, predisposition to cardiac arrhythmias, arterial vasodilation, impaired responsiveness to catecholamines, reduced hepatic blood flow, and impaired oxygen tissue delivery. Metabolic effects of acidosis include reduction of ATP synthesis in the cells, increased ionized calcium levels, and insulin resistance. In severe acidemia (blood pH < 7.1) these effects can result in organ dysfunction and contribute to increased morbidity and mortality. Treatment of lactic acidosis includes recognition and correction of the underlying cause, provision of adequate oxygen tissue delivery, and reduction of oxygen tissue demand through sedation and mechanical ventilation. In addition, although not recommended by published guidelines, ICU clinicians sometimes attempt to increase blood pH with administration of intravenous (IV) sodium bicarbonate. However, persistence of lactic acidosis despite appropriate therapy is associated with very high (up to 90%) mortality [21–24].

Metabolic acidosis can be the result of either a primary increase in hydrogen ion (H^+^) or reduction of bicarbonate concentration. In acute states of metabolic acidosis, respiratory compensation through hyperventilation results in relative reduction of PaCO_2_, whereas in chronic states the human body develops renal compensation through reabsorption of HCO_3_. The etiology of metabolic acidosis is divided into causes resulting in elevated anion gap (AG) and causes that do not. Lactic acidosis is a common cause of high anion gap metabolic acidosis [9, 25–27] and is classified as type A (anaerobic), which is associated with tissue hypoperfusion and denotes a condition of intact mitochondrial function but inadequate oxygen delivery (such as in shock states), or type B (aerobic), wherein oxygen supply is adequate and there is no tissue hypoxia, but acidosis is due to abnormal carbohydrate metabolism, such as in hepatic failure, diabetes, or phenformin intoxication [28, 29]. In patients with severe sepsis and septic shock, tissue hypoxia occurs due to reduction of oxygen delivery to peripheral tissues [30], while other mechanisms, including reduction of lactate clearance, reduced activation of the pyruvate dehydrogenase complex, and alterations of mitochondrial function contribute to this process.

Normal blood lactate levels in healthy humans are 0.5–1 mmol/L, but “normal” lactate concentrations in patients with critical illness are <2 mmol/L.* Hyperlactatemia* is defined as mild to moderate when persistent increase in blood lactate concentration (2–4 mmol/L) without metabolic acidosis exists, whereas* lactic acidosis* is characterized by persistently increased blood lactate levels (>4-5 mmol/L) in association with metabolic acidosis. Acidosis can inhibit lactic acid production by reducing activity of the enzyme phosphofructokinase; therefore correction of metabolic acidosis with sodium bicarbonate may increase lactic acid production by inhibiting this compensatory response [31–34].

Bicarbonate is the main form of CO_2_ in the human body and can be estimated from pH and pCO_2_. Normal pH range is 7.35–7.45 and normal bicarbonate is 21–28 mEq/L. Blood pH is reduced (acidosis) when CO_2_ quantity is elevated and increases (alkalosis) when CO_2_ quantity is reduced or when the quantity of base, such as bicarbonate (HCO_3_^−^), increases in blood. Bicarbonate is secreted and absorbed again (conservation) from the kidneys in response to pH changes and is commonly encountered as sodium bicarbonate (NaHCO_3_). Bicarbonate administration can stimulate superoxide formation, increase proinflammatory cytokine release, and enhance apoptosis and may also result in paradoxic intracellular acidosis due to generation of CO_2_. In clinical use, bicarbonate administration in severe academia may cause volume expansion and hypernatremia but can also result in reduced blood pressure and cardiac output and increased mortality.

If bicarbonate is to be used, it should be administered as slow infusion, and the amount of CO_2_ that is produced should be monitored. In patients with sepsis and severe acidosis undergoing renal replacement therapy, use of bicarbonate-based replacement fluids is recommended. Similarly, chronic bicarbonate replacement is indicated in patients with renal tubular acidosis.

Worldwide, many intensive care clinicians consider bicarbonate therapy as major component in the treatment of sepsis in patients with metabolic acidosis [35] and in sepsis patients with pH < 7.20, or, even more so, in patients with pH < 7.0. This therapeutic approach is supported by evidence suggesting that acute acidemia has deleterious effects on organ function. However, although acidemia correction with administration of bicarbonates aims to normalize extracellular and intracellular pH and improve outcome [11], several studies suggest that this approach is overly simplistic [36] and the impact of bicarbonate administration on restoring hemodynamics, reducing vasopressor requirements, and improving clinical outcomes is unknown [21]. In 2000, a comment published in “Chest” by Cuhaci in response to an earlier study by Forsythe and Schmidt [36] criticized the suggestion to not routinely use bicarbonates in lactic acidosis and questioned whether rejection of a reasonable therapeutic option is wise, when this option does not cause harm and there are no proven alternative options [37]. This comment likely reflects clinical practice, as evidenced by a survey published in 2006, which showed that 67% of 48 intensivists who responded to the survey would give bicarbonate for treatment of lactic acidosis [9]. However, the argument that bicarbonate administration does not cause harm is contradicted by the “surviving sepsis guidelines” which state that “Bicarbonate administration has been associated with sodium and fluid overload, an increase in lactate and PCO_2_, and a decrease in serum ionized calcium…” [21].

The appropriate treatment of metabolic acidosis, especially organic acidosis (such as lactic acidosis), is controversial, but reduction of acid production via improved oxygen tissue delivery is generally considered the most effective treatment. Aggressive therapy of sepsis is based on appropriate antibiotics and on addressing the causes of acidosis, with fluid resuscitation and restoration of cardiac function. Goals of resuscitation in sepsis with severe metabolic acidosis include maintenance of CVP > 8 mmHg, ScvO_2_ > 70% and normalization of lactate, while bicarbonate administration remains controversial [5].

Reasons for the absence of clear benefit from bicarbonate administration include exacerbation of intracellular acidosis due to initial rapid influx of CO_2_, loss of protective effects provided by acidosis against hypoxic damage, the fact that more calcium will bind to albumin instead of H^+^ leading to reduction of ionized calcium which, in turn, decreases cardiac output, and cellular swelling and dysfunction resulting from acceleration of cellular influx of sodium and calcium in response to worsening intracellular acidosis.

Additional risks of sodium bicarbonate administration include increased plasma PaCO_2_, hyperosmolality, hypernatremia, volume overload, and pH overcorrection resulting in metabolic alkalosis. Finally, regarding the amount of bicarbonate needed for correction of metabolic acidosis, there is no simple formula, because many confounding factors can affect acid-base status and bicarbonate volume of distribution. In general, bicarbonate deficit can be calculated using the equation “HCO_3_^−^ deficit = (desired HCO_3_^−^ − measured  HCO_3_^−^) × 0.5 × body weight” [33]. The sodium bicarbonate amount to be administered should be infused over hours, rather than as bolus [24, 36, 38, 39]. When decision to administer bicarbonates has been made, clinicians should monitor for adverse effects, facilitate elimination of excess CO_2_ produced, and correct hypocalcemia, because a 10% reduction of plasma calcium level can depress myocardial contractility and reduce vascular responsiveness to catecholamines [12].

Sodium bicarbonate increases plasma pH, but this effect is transient and does not correlate with hemodynamic improvement or improved outcomes in sepsis patients; therefore addressing the cause remains the most effective therapy for metabolic acidosis in sepsis. Although acidemia contributes to cardiovascular dysfunction in sepsis, no controlled study has demonstrated hemodynamic improvement with bicarbonate therapy, regardless of the effect on pH, and studies have shown worsening of the metabolic and hemodynamic consequences of acidosis [24, 40]. As there are no documented benefits from use of sodium bicarbonate in lactic acidosis related to inadequate tissue perfusion, routine use of sodium bicarbonate has been removed from the Advanced Cardiac Life Support algorithms [40]. In agreement with current guidelines, our literature search suggests that although there is debate regarding sodium bicarbonate use for treatment of lactic acidosis in sepsis, currently available evidence does not support the use of bicarbonate in sepsis patients with metabolic acidosis.

## 5. Conclusions

Routine bicarbonate administration for treatment of lactic acidosis in sepsis is subject to ongoing debate. Published evidence suggests that bicarbonate therapy is not beneficial in cases of metabolic acidosis in sepsis and may even cause harm by worsening intracellular acidosis. Rational treatment of metabolic acidosis in sepsis is directed towards addressing the underlying causes of acidosis and optimizing tissue oxygen delivery through optimization of cardiopulmonary parameters. However, as recent clinical data from China suggest that sodium bicarbonate therapy may actually improve outcome in sepsis patients with acidosis, large, rigorous, well-designed prospective clinical trials are needed to thoroughly assess the value of bicarbonate therapy in this patient population.

## Figures and Tables

**Table 1:** Clinical studies evaluating bicarbonate use in sepsis patients with acidosis. Studies are listed in chronological order, based on year of publication.

Reference	Country, year	Study design	Results
Cooper et al. [1]	British Columbia, Canada, 1990	Prospective, randomized, blinded, crossover study, 14 patients	Sodium bicarbonate did not improve hemodynamics or the response to catecholamines and caused hypocalcemia and hypercarbia

Mathieu et al. [2]	Lille, France, 1991	Prospective, randomized, blinded, crossover study, 10 patients	Sodium bicarbonate increased arterial and venous pH, serum bicarbonate, and arterial and venous blood pCO_2_, but hemodynamic responses similar to sodium chloride

Fang et al. [3]	Nanjing, China, 2008	Prospective, randomized trial, 94 patients	5% sodium bicarbonate for resuscitation in severe sepsis with hypotension improved blood pressure and cardiac output earlier than saline or hypertonic sodium chloride, indicating limited benefit from bicarbonate in sepsis

Noritomi et al. [4]	Sao Paulo, Brazil, 2009	Prospective, observational study, 60 ICU patients with severe sepsis or septic shock	Lactate reduced and metabolic acidosis corrected in survivors, but not in nonsurvivors

El-Sholh et al. [5]	New York, USA, 2010	Retrospective study, 36 patients with septic shock who received bicarbonates versus 36 matched patients who did not	Bicarbonate group had shorter duration of mechanical ventilation but no difference in 28-day mortality

Jung et al. [6]	Montpellier, France, 2011	Prospective multicenter observational study, 200 patients with severe acidosis	Bicarbonate administration in 5–55% of patients, depending on center, not on acidosis mechanism. No association between bicarbonate and outcome

Chen et al. [8]	Jiangsu, China, 2013	Prospective, randomized trial, 65 patients	Patients who received bicarbonate had improved hemodynamics, shorter mechanical ventilation, shorter ICU and hospital stay, and lower mortality

Kim et al. [7]	Busan, Korea, 2013	Retrospective study, 103 patients with lactic acidosis	Bicarbonate use was independent risk factor for increased mortality

**Table 2:** Laboratory studies evaluating bicarbonate administration in sepsis or acidosis. Studies are listed in chronological order, based on year of publication.

Reference	Country, year	Study design	Results
Garella et al. [14]	Providence, RI, USA, 1973	156 dogs, low, normal, or high bicarbonate concentration	Preexisting plasma bicarbonate concentration influences the apparent bicarbonate volume of distribution

Arieff et al. [16]	San Francisco, USA, 1982	Dogs with phenformin-induced lactic acidosis	Bicarbonate reduced portal vein blood flow, cardiac output, and pH in liver and erythrocytes

Benjamin et al. [17]	New York, USA, 1994	Experimental hemorrhagic shock in dogs, compared bicarbonate versus hypertonic saline versus Carbicarb	Bicarbonate moderately increased blood pressure, cardiac index, oxygen delivery, and oxygen consumption but not better than hypertonic saline

Goldsmith et al. [18]	London, UK, 1997	Human leukocytes in vitro	Sodium bicarbonate caused intracellular acidification

Levraut et al. [19]	Nice, France, 2001	Human hepatocytes in vitro	Sodium bicarbonate decreases intracellular pH, and intracellular pH changes are linked to pCO_2_ in surrounding fluid

Nielsen et al. [20]	Copenhagen, Denmark, 2002	31P-magnetic resonance spectroscopy in skeletal muscle of healthy subjects	Bicarbonate administration attenuated the reduction of intracellular muscle pH

Valenza et al. [15]	Milan, Italy, 2012	32 Sprague Dawley rats in four groups	Bicarbonate corrected acidosis in normoxic, normotensive rats, but effects of bicarbonate on pH different in rats receiving transient versus persistent lactic acid infusion
